# Characterization of *Salmonella* serotypes prevalent in asymptomatic people and patients

**DOI:** 10.1186/s12879-021-06340-z

**Published:** 2021-07-01

**Authors:** Haiyan Xu, Weibing Zhang, Kai Zhang, Yue Zhang, Zhenyu Wang, Wei Zhang, Yang Li, Qiuchun Li

**Affiliations:** 1 Nantong Center for Disease Control and Prevention, Nantong, China; 2 Key Laboratory of Prevention and Control of Biological Hazard Factors (Animal Origin) for Agri-food Safety and Quality, Ministry of Agriculture of China, Yangzhou University, Yangzhou, China; 3 Jiangsu Key Laboratory of Zoonosis/Jiangsu Co-Innovation Center for Prevention and Control of Important Animal Infectious Diseases and Zoonoses, Yangzhou University, Yangzhou, China; 4 Joint International Research Laboratory of Agriculture and Agri-Product Safety, Yangzhou University, Yangzhou, China

**Keywords:** *Salmonella*, Asymptomatic infection, PFGE, Antimicrobial susceptibility

## Abstract

**Background:**

Infection with *Salmonella enterica* usually results in diarrhea, fever, and abdominal cramps, but some people become asymptomatic or chronic carrier as a source of infection for others. This study aimed to analyze the difference in serotype, antimicrobial resistance, and genetic profiles between *Salmonella* strains isolated from patients and those from asymptomatic people in Nantong city, China.

**Methods:**

A total of 88 *Salmonella* strains were collected from patients and asymptomatic people from 2017 to 2018. Serotyping, antimicrobial susceptibility testing, and PFGE analysis were performed to analyze the characteristics of these strains.

**Results:**

Twenty serotypes belonging to 8 serogroups were identified in the 88 *Salmonella* strains. *S.* Typhimurium remained to be the predominant serotype in strains from both patients and asymptomatic people. Among the 27 strains from patients, *S*. Enteritidis and *S.* Rissen were shown as the other two major serotypes, while *S.* London, *S.* Derby, and *S.* Meleagridis were demonstrated as the other significant serotypes among the 61 strains from asymptomatic people. Antimicrobial resistance testing revealed that 84.1% of strains from both resources were multi-drug resistant. PFGE displayed a highly discriminative ability to differentiate strains belonging to *S.* Derby, *S.* Typhimurium, etc., but could not efficiently differentiate serotypes like *S.* Enteritidis.

**Conclusions:**

This study’s results demonstrated that *S.* Typhimurium could cause human infection in both symptomatic and asymptomatic state; *S*. London, *S*. Derby, and *S*. Meleagridis usually cause asymptomatic infection, while *S*. Enteritidis infection mainly results in human diseases. The high multi-drug resistance rate detected in the antimicrobial resistance and diverse PFGE profiles of these strains implied that the strains were isolated from different sources, and the increased surveillance of *Salmonella* from both patients and asymptomatic people should be taken to control the disease.

**Supplementary Information:**

The online version contains supplementary material available at 10.1186/s12879-021-06340-z.

## Background

Salmonellosis is an infection with bacteria called *Salmonella*. Human salmonellosis generally manifests two kinds of disorders: typhoid fever caused by typhoidal *Salmonella enterica* serotypes *S.* Typhi, *S.* Paratyphi A, *S.* Paratyphi B, and *S.* Paratyphi C, and another is gastroenteritis caused by nontyphoidal *Salmonella* (NTS) serotypes such as *S.* Enteritidis and *S.* Typhimurium [1]. *Salmonella* can cause symptomatic infections, which is defined as the occurrence of any number of watery stools during 24 h period accompanied by fever, vomiting or abdominal cramps [2]. Besides, some people can get a *Salmonella* infection without any symptoms and clear the infection within a few days, or become persistent carrier of the bacteria, which are described as asymptomatic or chronic infection, respectively [3]. A bacterial load of 10^6^–10^8^ CFU of NTS organisms is needed to cause symptomatic disease in healthy adults [4]. It is estimated that approximately 93.8 million gastroenteritis cases and 155,000 deaths are attributed to NTS worldwide annually [5]. According to the United States and European Food Safety Authority (EFSA) data, *S.* Enteritidis and *S*. Typhimurium have remained the top serotypes causing clinical human salmonellosis [6, 7]. Both serotypes were predominantly contributed to human gastroenteritis due to *Salmonella* infection in hospitals from different provinces or cities of China [8–11]. With difference to the serotypes identified in patients, *S.* Derby, *S.* London, and *S.* Senftenberg have been demonstrated to be the major *Salmonella* serotypes in asymptomatic food handlers as well as *S.* Typhimurium and *S.* Enteritidis [12]. Therefore, it is necessary to characterize the difference in *Salmonella* serotypes between patients and asymptomatic people.

Phenotypic and genotypic methods allow the identification and characterization of bacterial strains with different sources [13]. *Salmonella* serovars’ appearance with multidrug-resistant (MDR) patterns has increased rapidly and become a heavy burden on the clinical treatment of salmonellosis [4, 14]. A report of 1826 NTS isolates from human patients in Guangdong province of China revealed that 46% of the isolates were MDR, and 72% showed resistance to at least one antimicrobial [8]. Among 109 *Salmonella* isolates from diarrheagenic children in Beijing, 50% of the strains showed resistance to at least three antimicrobials, and 12.8% were resistant to six [15]. In Malaysia, seven *Salmonella* strains were isolated from a total of 317 asymptomatic food handlers, and these strains showed multidrug-resistance to ampicillin, chloramphenicol, trimethoprim-sulfamethoxazole, sulfonamides, streptomycin, and tetracycline [16]. Since the bacteria have evolved to be resistant to various antimicrobials, constant antibiotic surveillance is warranted, and the genotyping techniques are essential to tracing the source of the bacteria. The pulsed-field gel electrophoresis (PFGE) was considered as the golden standard genotyping technique for differentiation of *Salmonella* isolates [17, 18]. Furthermore, it can also be used to trace the source of infections and the transmission route of the strains [18].

This study investigated the serotypes distribution, antimicrobial resistance, and PFGE profiles of *S. enterica* isolated from patients and asymptomatic people in Nantong, China. By comparing the characteristics of *Salmonella* strains from two different kinds of sources, we could develop effective strategies to control *Salmonella* infection in humans.

## Methods

### Serotyping of *Salmonella* isolates from human

A total of 88 *Salmonella* isolates were obtained from patients and asymptomatic people by the Center for Disease Control and Prevention, Nantong, China. Among the 88 strains, 61 was isolated from people on physical examination without any symptoms, while 27 was isolated from patients with diarrhea. This study received ethical approval from the Ethics Committees of Center for Disease Control and Prevention of Nantong city. Serotyping of the isolates was performed using slide agglutination test according to the instructions of *Salmonella* antisera kit (Tianrun Bio-Pharmaceutical Co. Ltd., Ningbo, China) based on somatic O, as well as phase 1 and phase 2 flagella antigens. The serotype identification of each strain was based on the Kauffmann-White scheme [19].

### Antimicrobial susceptibility testing

Susceptibility testing of the 88 *Salmonella* isolates with the Sensititre National Antimicrobial Resistance Monitoring System Gram-negative susceptibility plates (Customized version, Sensititre; Trek Diagnostic Systems, Inc., Westlake, OH) was performed according to the manufacturer’s instructions. Twenty-six antimicrobial agents (antimicrobial abbreviations and dilution concentration ranges are given in parentheses, in micrograms per milliliter) were used as follows: ampicillin (AMP, 2–64); ampicillin-sulbactam (AMS, 2–64 and 1–32); amoxicillin-clavulanic acid (AMC, 2–64 and 1–32); aztreonam (AZM, 1–32); cefazolin (CFZ, 0.5–16); cefotaxime (CTX, 0.25–8); ceftazidime (CAZ, 0.5–16); cefoxitin (CFX, 2–64); cefepine (FEP, 0.25–16); gentamicin (GEN, 1–32); amikacin (AMI, 1–32); imipenem (IMI, 0.25–8); meropenem (MEM, 0.06–4); chloramphenicol (CHL, 2–64); trimethoprim/sulfamethoxazole (SXT, 0.25–8 and 4.75–152); sulfisoxazole (SUL, 32–512); tetracycline (TET, 1–32); minocycline (MIN, 1–32); nalidixic acid (NAL, 4–64); ciprofloxacin (CIP, 0.03–32); levofloxacin (LEV, 0.125–8); doxycycline (DOX, 0.5–16); kanamycin (KAN, 8–64); streptomycin (STR, 4–32); colistin sulphate (CT, 0.5–16); polymyxin B (PB, 0.5–16); The Clinical and Laboratory Standards Institute (CLSI, 2018) on Antimicrobial Susceptibility Testing breakpoints were used to assess the results [20]. *Escherichia coli* ATCC25922 with known antimicrobial resistance profiles was used as a quality control organism.

### PFGE

PFGE was conducted to reveal the clonal-relatedness of *Salmonella *strains following the standardized laboratory protocol for molecular subtyping of *Salmonella* by PFGE [17]. The *S.* Braenderup H9812 was used as a marker strain. Briefly, agarose-embedded genomic DNA samples were digested with *Xba*I (Takara, Japan) at 37 °C for 2 h. The Chef Mapper electrophoresis system was then used to separate restriction fragments in 0.5X Tris-borate- ethylenediaminetetraacetic acid (EDTA; TBE) extended-range buffer (Bio-Rad, United States) with recirculation at 14 °C for 18–20 h. The gel was stained with GelRed and visualized under UV light to record the gel results with TIFF images. The genetic patterns for each of the strains were compared and analyzed using the BioNumericus version 7.5 software (Applied Maths, Belgium). A dendrogram was produced using the Dice coefficient correlation and unweighted pair group method using the arithmetic mean algorithm (UPGMA) with 1.5% optimization and a band position tolerance.

## Results

### Prevalence and distribution of serotypes for *Salmonella* isolates from humans

**Fig. 1:**
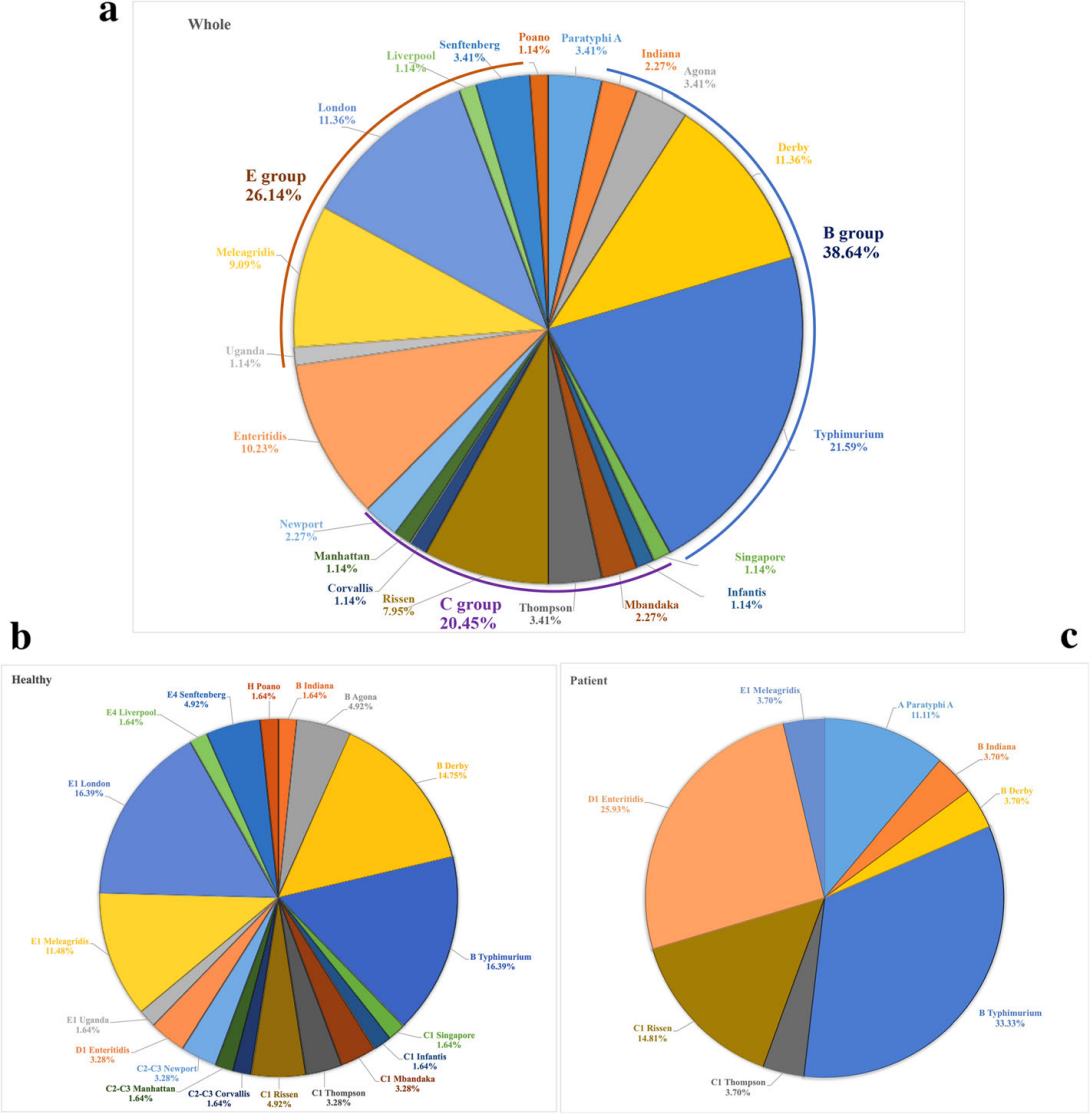
Prevalence of *Salmonella* serotypes in humans. The distribution of *Salmonella* serotypes among 88 isolates from humans (**a**), including 61 isolates from asymptomatic people (**b**) and 27 isolates from patients (**c**). The B group, C group, and E group represents *Salmonella* serogroup B (O:4), C [C_1_(O:7); C_2_-C_3_(O:8)], E [E_1_(O:3,10); E_4_ (O:1,3,19)], respectively

A total of 88 *Salmonella* strains were isolated, 49 (55.7%) from males and 39 (44.7%) from females in Nantong city (Table 1). Nearly 69.3% (61/88) of strains were isolated from asymptomatic people, while 30.7% (27/88) were from patients (Table 1). Among the 27 strains from patients, 8 (29.6%) were isolated from children. All of the strains with their information, including the age, sex, and samples, were listed in Table S1. Among the identified 88 *Salmonella* isolates, 20 serotypes distributed in 8 serogroups were identified, with B (38.6%) and E (26.1%) as the top 2 predominant serogroups (Table 2). The *S.* Typhimurium serotype was detected in 21.6% of the 88 isolates, followed by 12.5% of *S.* London, 11.4% of *S.* Derby, 10.2% of *S.* Enteritidis, and 7.5% of both *S.* Rissen and *S.* Meleagridis (Table 2, Fig. 1a). However, compared to 19 serotypes identified in 61 isolates from asymptomatic people, 8 serotypes were detected in 27 isolates from patients (Table 2, Fig. 1b). Only 1 out of 11 *S.* London isolates was obtained from a patient, while 77.8% (7/9) of *S.* Enteritidis can cause human disease, and all of the 3 *S.* Paratyphi A strains were isolated from patients (Table 2, Fig. 1c). *S* Typhimurium (16.4%) and *S*. London (16.4%) was the predominant serotype in the isolates from asymptomatic people, followed by *S.* Derby (14.8%) and *S.* Meleagridis (11.5%) (Fig. 1b). In contrast, *S*. Typhimurium (33.3%) and *S*. Enteritidis (25.9%) were the top 2 serotypes causing symptoms in patients, followed by *S.* Rissen (14.8%) (Fig. 1c).

**Table 1:** The sampling information of *Salmonella* from humans

Variable	Value
Sex	n^a^ (%)
Male	49 (55.7%)
Female	39 (44.3%)
Age	median (range)
	37 (1–77)
Place	n (%)
Nantong CDC	61 (69.3%)
Chongchuan district	23 (26.1%)
Other counties	4 (4.6%)
Source	n (%)
Asymptomatic people	61 (69.3%)
Patient	27 (30.7%)

^a^n represents the number of strains

**Table 2:** Distribution of serotypes for 88 *Salmonella* isolates from patients and asymptomatic people

O group (no)	Serotype (no, percentage^a^)	NO.	Percentage (%)
A (3)	Paratyphi A (3, 100%)	3	3.4
B (34)	Indiana (1, 50%)	2	2.3
	Agona	3	3.4
	Derby (1, 10%)	10	11.4
	Typhimurium (9, 47.4%)	19	21.6
C1 (14)	Singapore	1	1.1
	Infantis	1	1.1
	Mbandaka	2	2.3
	Thompson (1, 33.3%)	3	3.4
	Rissen (4, 57.1%)	7	8.0
C2-C3 (4)	Corvallis	1	1.1
	Manhattan	1	1.1
	Newport	2	2.3
D1 (9)	Enteritidis (7, 77.8%)	9	10.2
E1 (19)	Uganda	1	1.1
	Meleagridis (1, 12.5%)	8	9.1
	London	10	11.4
E4 (4)	Liverpool	1	1.1
	Senftenberg	3	3.4
H (1)	Poano	1	1.1

^a^represents the number of strains isolated from patients with diarrhea, fever, or abdominal cramps in hospitals and the percentage of these strains among the same serotype

### Antimicrobial resistance

**Fig. 2:**
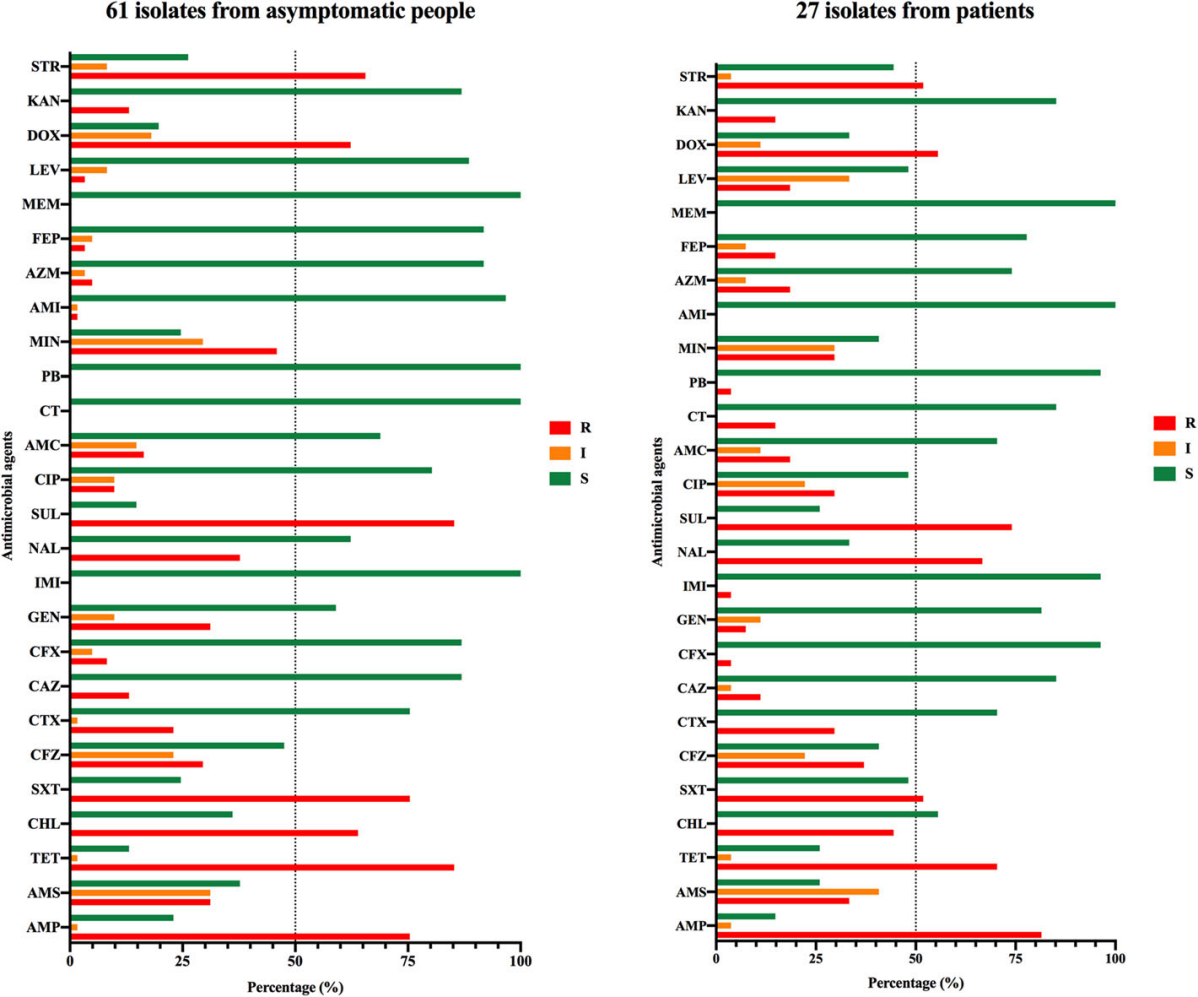
Antimicrobial resistance of human *Salmonella* isolates. The percentage of strains with resistance to each antimicrobial agent. The “R” displayed as red column represents resistant, the “I” displayed as orange column represents intermediate, and the “S” displayed as green column represents susceptible

A total of 8 (8/88, 9.1%) strains were susceptible to all the 26 antimicrobials, and all of the 88 isolates showed susceptibility to meropenem, a type of carbapenems used to treat symptomatic *Salmonella* infection (Fig. 2). *S.* Derby showed a high rate of multi-drug resistance to these antimicrobials (90%, 9/10), followed by *S.* Enteritidis (88.9%, 8/9), *S.* Meleagridis (85.7%, 6/7), *S.* Typhimurium (84.2%, 16/19), *S.* London (81.8%, 9/11), and *S.* Rissen (71.4%, 5/7). Besides, 85.2% (52/61) of the isolates from asymptomatic humans and 85.2% (23/27) of those from patients were identified to be MDR (Fig. 2).

Among the 26 antimicrobial agents belonging to 8 classes (β-Lactamases, aminoglycosides, carbapenems, polymyxins, phenicols, sulfonamides, tetracyclines, fluoroquinolones), resistance to sulfisoxazole, tetracycline, and ampicillin was found in 81.8, 80.7, 77.3% of isolates, respectively (Fig. 2). These top three antimicrobials were also seen in the 61 isolates from asymptomatic people. Besides, 73.8% (45/61) of these isolates showed resistance to the three antimicrobials (Fig. 2). However, ampicillin (81.5%), tetracycline (70.4%), and nalidixic acid (66.8%) were the top three antimicrobials found in 27 isolates from patients. Resistance to all of the three antimicrobials was seen in 51.9% (14/27) of these isolates.

Among the 88 isolates, the *S.* Meleagridis F2–6 and *S.* Rissen F5–5 strain showed resistance to amikacin and imipenem, respectively (Fig. 2). Four isolates belonging to three serotypes (*S.* Parytyphi A, *S.* Enteritidis, *S.* Typhimurium) were resistant against polymyxin E, an antibiotic medication used as a last-resort treatment for MDR gram-negative infections, and all four strains were isolated from patients. The *S.* Enteritidis F6–1 strain displayed resistance to both polymyxin E and polymyxin B.

### PFGE analysis

**Fig. 3:**
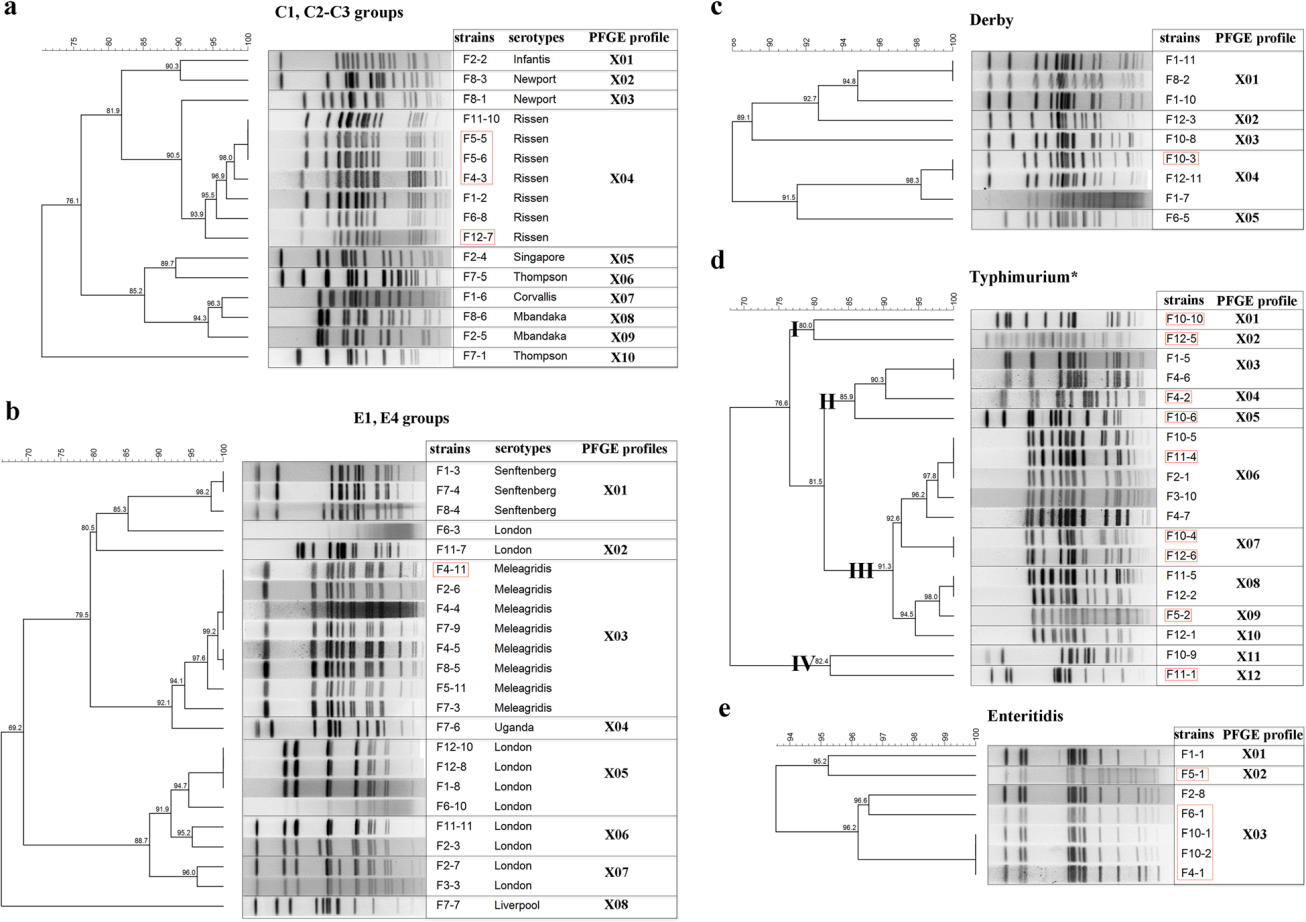
PFGE analysis of *Salmonella* strains. PFGE profiles and phylogenic relationship of strains belonging to C_1_ and C_2_-C_3_ groups (**a**), E_1_ and E_4_ groups (**b**), *S.* Derby (**c**), *S.* Typhimurium (**d**), and *S.* Enteritidis (**e**). The strains in red box were isolated from patients. The “I”, “II”, “III”, and “IV” represents the four clusters of *S.* Typhimurium (including *S.* Typhimurium monophasic variants) divided by PFGE analysis

The 88 strains were then subjected to the PFGE analysis with the restriction enzyme *Xba*I for inter-serotype differentiation to reveal their genetic relationship. Among the 16 strains belonging to 8 serotypes in C1 and C2-C3 groups, 10 profiles (X01 to X10) were identified with X04 shared by all of the 8 *S.* Rissen strains isolated from both patients and asymptomatic people (Fig. 3a). Among the 23 strains belonging to E1 and E4 groups, 8 genetic profiles (designated X01 to X08) were presented to show the genetic difference of 6 serotypes (Fig. 3b). The X01 and X03 represented 3 *S.* Senftenberg and 8 *S.* Meleagridis strains, respectively (Fig. 3b). Except for one strain of *S.* London without clear DNA bands, the other 9 strains were distributed in 4 different profiles, which were X02, X05, X06, and X07 (Fig. 3b).

Among the 34 strains belonging to the B group, *S.* Typhimurium and *S.* Derby took up 85.3% with diverse PFGE profiles (Fig. 3c, d). Twelve profiles (X01 to X12) were identified in 19 *S.* Typhimurium strains (Fig. 3d). The X01, X02, X04, X05, X07, X019, and X12 profiles were detected in strains isolated from patients, while the X03, X08, X10, and X11 profiles were found in strains isolated from asymptomatic people (Fig. 3d). The X06 profile consists of five strains from both sources, but 80% (4/5) of them in this profile were from asymptomatic people (Fig. 3d). Except for one strain of *S.* Derby with smear patterns, the other 9 strains displayed 5 PFGE profiles (X01 to X05) with X01 and X04 as predominant profiles, each of which are shared by 3 strains (Fig. 3c). The only one strain F10–3 isolated from the patient belonged to the X04 genetic profile (Fig. 3c). In our study, *S.* Enteritidis is the only detected serotype in the D group. Three profiles (X01, X02, and X03) were presented for 8 out of 9 *S.* Enteritidis strains. Four strains (F6–1, F10–1, F10–2, and F4–1) belonging to the X03 were obtained from patients, and X03 was identified as the predominant PFGE profile (6/8, 75%) for the 8 strains (Fig. 3e).

## Discussion

### Distribution of *Salmonella* spp. in patients and asymptomatic people

*Salmonella* is one of the major pathogens causing human diarrhea, which is closely related to the consumption of bacterially contaminated foods. Therefore, *Salmonella* reports have been published every year by the US National Enteric Disease Surveillance system, the European Food Safety Authority (EFSA), and the European Centre for Disease Prevention and Control (ECDC). According to the US and EU reports, *S.* Enteritidis and *S.* Typhimurium (including *S.* Typhimurium monophasic variants) have been the top 2 serotypes causing human salmonellosis [6, 7]. Our study showed that *S.* Typhimurium is the predominant serotype from diarrhea patients in Nantong city, followed by *S.* Enteritidis, correspondent to the previous reports in China [8]. However, in asymptomatic people, *S.* London became the predominant serotype as well as *S.* Typhimurium, followed by *S.* Derby and *S.* Meleagridis. The difference in serotype distribution in patients and asymptomatic people reflected that many NTS serotypes could infect humans without any symptom, but these serotypes were underestimated in the existing surveillance system for mostly patients. Additionally, these NTS serotypes have frequently been isolated from pig, chicken, and their associated meat products [14, 21], implying the potential transmission of *Salmonella* from animal foods to humans. Except for *S.* Typhimurium causing disease or no symptoms, *S.* Rissen displayed a similar characteristic to *S.* Typhimurium in human infection (Table 2). In 2009, *S.* Rissen caused > 80 people infection in over 4 different states of the USA, and the cases for human infection by *S.* Rissen were also reported in Denmark, Thailand, UK, and China [22–24]. Among 208 *S.* Rissen isolates from human samples, 108 out of the isolates were from patients, 100 isolates were from the asymptomatic carriers [22], reflecting that nearly 50% of the people infected with *S.* Rissen were in the asymptomatic state as well as in our study (Table 2). However, some serotypes have been mainly isolated from patients, such as the typhoidal *S.* Paratyphi A (100%) obtained from blood samples, and nontyphoidal *S.* Enteritidis (77.8%) collected from diarrheagenic patients.

### Multidrug-resistance of *Salmonella* spp.

Among the used 26 antimicrobial agents belonging to 8 different types, 84.1% (74/88) of *Salmonella* isolates showed resistance to at least three types of antimicrobials, which is dramatically higher than the reported 46 and 50% of *Salmonella* isolates from patients were MDR in Guangdong and Beijing, respectively [8, 15]. Among the 61 *Salmonella* isolates from asymptomatic people, 49.2% (30/61) of the strains showed resistance to 6 out of 8 types of antimicrobial agents, while 73.8% showed resistance to 5 types of antimicrobials. This demonstrated that the NTS organisms from asymptomatic people showed strong resistance to antimicrobials as well as the human diarrheal or bloodborne isolates, which is similar to the report that 81.8% of *Salmonella* isolates from asymptomatic food handlers were MDR [25]. However, the result was different from the recently reported fewer MDR NTS isolates in asymptomatic children than in symptomatic individuals in Vietnamese [26]. Twenty-three out of 88 *Salmonella* isolates showed resistance to one or more antimicrobial agents belonging to extended-spectrum β-lactamases (ESBLs). One ESBL *S.* Thompson strain displayed resistance to all of the detected β-lactamases, including aztreonam, cefepime, cefazolin, ceftazidime, and amoxicillin/clavulanic acid. The emergence of ESBL-producing *Salmonella* may cause a substantial increase in treatment costs and prolonged treatment periodicity [27].

### PFGE differentiation of *Salmonella* strains

Although the whole-genome sequencing (WGS)-based typing methods have been considered highly discriminative epidemiological tools, PFGE has a relatively high concordance with epidemiological relatedness [28]. The PFGE profiles not only showed perfect correspondence to serotypes belonging to C1, C2–3 or E1, E4 serogroups (Fig. 3a, b), but also displayed a highly discriminative ability to different strains of serotypes like *S.* London, *S.* Newport, and *S.* Mabandaka, which is potentially caused by the integration of new genetic elements for adaption to adverse conditions [29].

*S.* Derby is another frequently reported serotype isolated from both human and animal or animal foods. Most isolates were obtained from asymptomatic people, revealing that it is not the predominant serotype causing severe infections in humans. In this study, the 9 *S*. Derby strains were divided into 5 profiles by PFGE analysis, which has been confirmed as a molecular subtyping method used to differentiate *S.* Derby strains (Fig. 3c). Ten PFGE profiles were obtained in 16 *S.* Derby strains of human origin with 9 antimicrobial resistance patterns [30]. With difference to *S.* Derby, *S.* Typhimurium is the predominant serotype causing either human diarrhea or asymptomatic infection [30]. Four clusters were identified in 19 *S.* Typhimurium strains, most of which are distributed in cluster III, including 7 and 4 strains from asymptomatic people and patients, respectively (Fig. 3d). This is the reason for considering *S.* Typhimurium as one of the most important serotypes in the National *Salmonella* surveillance system [6]. Another serotype, *S.* Enteritidis has been confirmed as the predominant serotype causing human diarrhea, and it could not be efficiently differentiated by PFGE (Fig. 3e). Other molecular typing methods, such as CRISPR typing and whole-genome sequencing (WGS) based typing, can be further used to study the evolutionary relationship of these isolates [31, 32].

Although PFGE has been considered as the “gold standard” for bacterial typing, the WGS has superior resolution to PFGE, and it can differentiate isolates which were indistinguishable by PFGE. WGS can distinguish stains with difference at only a single nucleotide and provide higher resolution than the other molecular typing methods [33]. In this study, the PFGE showed high discriminatory power in some serotypes, such as *S*. Typhimurium, but it could not efficiently distinguish *S.* Enteritidis isolates. Further analysis will be performed to reveal the relationship of the isolates belonging to the same serotype from different sources.

## Conclusion

This study compared the serotypes, antimicrobial resistance phenotypes, and genetic profiles of *Salmonella* strains between asymptomatic people and patients. The results revealed that *S*. Typhimurium is the predominant serotype causing human infection in both symptomatic and asymptomatic state. The other NTS including *S.* London, *S.* Derby, and *S.* Meleagridis mainly cause asymptomatic infection, while *S.* Enteritidis infection commonly results in human diseases. The high multi-drug resistance rate detected in these strains and diverse PFGE profiles showed no significant difference in strains between symptomatic and asymptomatic individuals, implying that all human-related *Salmonella* strains can induce both human salmonellosis and asymptomatic infection. Therefore, increased surveillance of *Salmonella* from both patients and asymptomatic people should be taken to control the transmission of the pathogen.

## Supplementary Information

**Additional file 1: Supplementary Table 1.** The information and antimicrobial resistance phenotype of human *Salmonella* isolates.

## Data Availability

The datasets used and/or analysed during the current study are available from the corresponding author on reasonable request.

## Abbreviations

NTS: Nontyphoidal *Salmonella*
MDR: Multidrug-resistant
EFSA: European Food Safety Authority
PFGE: Pulsed-field gel electrophoresis
CFU: Colony-forming unit
AMP: Ampicillin
AMS: Ampicillin-sulbactam
AMC: Amoxicillin-clavulanic acid
AZM: Aztreonam
CFZ: Cefazolin
CTX: Cefotaxime
CAZ: Ceftazidime
CFX: Cefoxitin
FEP: Cefepine
GEN: Gentamicin
AMI: Amikacin
IMI: Imipenem
MEM: Meropenem
CHL: Chloramphenicol
SXT: Trimethoprim/sulfamethoxazole
SUL: Sulfisoxazole
TET: Tetracycline
MIN: Minocycline
NAL: Nalidixic acid
CIP: Ciprofloxacin
LEV: Levofloxacin
Dox: Doxycyclin
KAN: Kanamycin
STR: Streptomycin
CT: Colistin sulphate
PB: Polymyxin B
CLSI: Clinical and Laboratory Standards Institute
ATCC: American Type Culture Collection
ECDC: The European Centre for Disease Prevention and Control
CRISPR: Clustered regularly interspaced short palindromic repeats
WGS: The Whole-genome sequencing
ESBLs: Extended-spectrum β-lactamases
UPGMA: The arithmetic mean algorithm
UV: Ultraviolet

## References

[1] Harrois D, Breurec S, Seck A, Delaune A, Le Hello S, Pardos de la Gandara M (2014). Prevalence and characterization of extended-spectrum β-lactamase-producing clinical *Salmonella enterica* isolates in Dakar, Senegal, from 1999 to 2009. Clin Microbiol Infect.

[2] Jertborn M, Haglind P, Iwarson S, Svennerholm AM (1990). Estimation of symptomatic and asymptomatic *Salmonella* infections. Scand J Infect Dis.

[3] Paudyal N, Pan H, Wu B, Zhou X, Zhou X, Chai W (2020). Persistent asymptomatic human infections by *Salmonella enterica* serovar Newport in China. mSphere.

[4] Chen HM, Wang Y, Su LH, Chiu CH (2013). Nontyphoid *Salmonella* infection: microbiology, clinical features, and antimicrobial therapy. Pediatr Neonatol.

[5] Majowicz SE, Musto J, Scallan E, Angulo FJ, Kirk M, O'Brien SJ, Jones TF, Fazil A, Hoekstra RM, International Collaboration on Enteric Disease 'Burden of Illness' Studies (2010). The global burden of nontyphoidal *Salmonella* gastroenteritis. Clin Infect Dis.

[6] Centers for Disease Control and Prevention (2018). National *Salmonella* surveillance annual report, 2016.

[7] European Food Safety Authority (2018). The European Union summary report on trends and sources of zoonoses, zoonotic agents and food-borne outbreaks in 2017. EFSA J.

[8] Liang Z, Ke B, Deng X, Liang J, Ran L, Lu L, He D, Huang Q, Ke C, Li Z, Yu H, Klena JD, Wu S (2015). Serotypes, seasonal trends, and antibiotic resistance of non-typhoidal *Salmonella* from human patients in Guangdong province, China, 2009-2012. BMC Infect Dis.

[9] Ke Y, Lu W, Liu W, Zhu P, Chen Q, Zhu Z (2020). Non-typhoidal *Salmonella* infections among children in a tertiary hospital in Ningbo, Zhejiang, China, 2012-2019. PLoS Negl Trop Dis.

[10] Li Y, Xie X, Xu X, Wang X, Chang H, Wang C, Wang A, He Y, Yu H, Wang X, Zeng M (2014). Nontyphoidal *Salmonella* infection in children with acute gastroenteritis: prevalence, serotypes, and antimicrobial resistance in Shanghai, China. Foodborne Pathog Dis.

[11] Ran L, Wu S, Gao Y, Zhang X, Feng Z, Wang Z, Kan B, Klena JD, Lo Fo Wong DMA, Angulo FJ, Varma JK (2011). Laboratory-based surveillance of nontyphoidal *Salmonell*a infections in China. Foodborne Pathog Dis.

[12] Xu H, Zhang W, Guo C, Xiong H, Chen X, Jiao X, Su J, Mao L, Zhao Z, Li Q (2019). Prevalence, serotypes, and antimicrobial resistance profiles among *Salmonella* isolated from food catering workers in Nantong, China. Foodborne Pathog Dis.

[13] Scott TM, Rose JB, Jenkins TM, Farrah SR, Lukasik J (2002). Microbial source tracking: current methodology and future directions. Appl Environ Microbiol.

[14] Xu C, Ren X, Feng Z, Fu Y, Hong Y, Shen Z, Zhang L, Liao M, Xu X, Zhang J (2017). Phenotypic characteristics and genetic diversity of *Salmonella enterica* serotype Derby isolated from human patients and foods of animal origin. Foodborne Pathog Dis.

[15] Qu M, Lv B, Zhang X, Yan H, Huang Y, Qian H, Pang B, Jia L, Kan B, Wang Q (2016). Prevalence and antibiotic resistance of bacterial pathogens isolated from childhood diarrhea in Beijing, China (2010-2014). Gut Pathog.

[16] Woh PY, Thong KL, Behnke JM, Lewis JW, Zain SNM (2017). Characterization of nontyphoidal *Salmonella* isolates from asymptomatic migrant food handlers in peninsular Malaysia. J Food Prot.

[17] Centers for Disease Control and Prevention (2017). The CDC PulseNet one-day (24–28 h) standardized laboratory protocol for molecular subtyping of *Escherichia coli* O157:H7, non-typhoidal *Salmonella* serotypes, and *Shigella sonnei* by Pulsed Field Gel Electrophoresis (PFGE).

[18] Diaz-Torres O, Lugo-Melchor OY, de Anda J, Gradilla-Hernandez MS, Amezquita-Lopez BA, Meza-Rodriguez D (2020). Prevalence, distribution, and diversity of *Salmonella* strains isolated from a subtropical lake. Front Microbiol.

[19] Grimont PA, Weill FX (2007). Antigenic formulae of the *Salmonella* serovars.

[20] Clinical and Laboratory Standards Institute (2018). Performance standards for antimicrobial susceptibility testing.

[21] Ma S, Lei C, Kong L, Jiang W, Liu B, Men S, Yang Y, Cheng G, Chen Y, Wang H (2017). Prevalence, antimicrobial resistance, and relatedness of *Salmonella* isolated from chickens and pigs on farms, abattoirs, and markets in Sichuan province, China. Foodborne Pathog Dis.

[22] Xu X, Biswas S, Gu G, Elbediwi M, Li Y, Yue M (2020). Characterization of multidrug resistance patterns of emerging *Salmonella enterica* serovar Rissen along the food chain in China. Antibiotics (Basel).

[23] Hendriksen RS, Bangtrakulnonth A, Pulsrikarn C, Pornreongwong S, Hasman H, Song SW, Aarestrup FM (2008). Antimicrobial resistance and molecular epidemiology of *Salmonella* Rissen from animals, food products, and patients in Thailand and Denmark. Foodborne Pathog Dis.

[24] Irvine N. Communicable diseases monthly report, Northern Ireland edition: The Health Protection Agency; 2009. Available online: http://www.publichealth.hscni.net/directorate-public-health/health-protection/surveillance-data. Accessed 15 Oct 2020.

[25] Solomon FB, Wada FW, Anjulo AA, Koyra HC, Tufa EG (2018). Burden of intestinal pathogens and associated factors among asymptomatic food handlers in South Ethiopia: emphasis on salmonellosis. BMC Res Notes.

[26] Parisi A, Le Thi Phuong T, Mather AE, Jombart T, Thanh Tuyen H, Phu Huong Lan N (2020). Differential antimicrobial susceptibility profiles between symptomatic and asymptomatic non-typhoidal *Salmonella* infections in Vietnamese children. Epidemiol Infect.

[27] Zhang SX, Zhou YM, Tian LG, Chen JX, Tinoco-Torres R, Serrano E, Li SZ, Chen SH, Ai L, Chen JH, Xia S, Lu Y, Lv S, Teng XJ, Xu W, Gu WP, Gong ST, Zhou XN, Geng LL, Hu W (2018). Antibiotic resistance and molecular characterization of diarrheagenic *Escherichia coli* and non-typhoidal *Salmonella* strains isolated from infections in Southwest China. Infect Dis Poverty.

[28] Tang S, Orsi RH, Luo H, Ge C, Zhang G, Baker RC, Stevenson A, Wiedmann M (2019). Assessment and comparison of molecular subtyping and characterization methods for *Salmonella*. Front Microbiol.

[29] Prosser JI, Bohannan BJ, Curtis TP, Ellis RJ, Firestone MK, Freckleton RP (2007). The role of ecological theory in microbial ecology. Nat Rev Microbiol.

[30] Valdezate S, Vidal A, Herrera-Leon S, Pozo J, Rubio P, Usera MA (2005). *Salmonella* Derby clonal spread from pork. Emerg Infect Dis.

[31] Li Q, Wang X, Yin K, Hu Y, Xu H, Xie X, Xu L, Fei X, Chen X, Jiao X (2018). Genetic analysis and CRISPR typing of *Salmonella enterica* serovar Enteritidis from different sources revealed potential transmission from poultry and pig to human. Int J Food Microbiol.

[32] Ktari S, Ksibi B, Ghedira K, Fabre L, Bertrand S, Maalej S (2020). Genetic diversity of clinical *Salmonella enterica* serovar Typhimurium in a university hospital of south Tunisia, 2000–2013. Infect Genet Evol.

[33] Salipante SJ, SenGupta DJ, Cummings LA, Land TA, Hoogestraat DR, Cookson BT (2015). Application of whole-genome sequencing for bacterial strain typing in molecular epidemiology. J Clin Microbiol.

